# Phytotoxicity of Chemical Compounds from *Cinnamomum camphora* Pruning Waste in Germination and Plant Cultivation

**DOI:** 10.3390/ijerph191811617

**Published:** 2022-09-15

**Authors:** Hong Wang, Wei Lin, Dongdong Zhang, Rui Yang, Wanlai Zhou, Zhiyong Qi

**Affiliations:** 1 Institute of Urban Agriculture, Chinese Academy of Agricultural Sciences, Chengdu 610213, China; 2 Biogas Institute of Ministry of Agriculture and Rural Affairs, Chengdu 610041, China; 3 Chengdu National Agricultural Science and Technology Center, Chengdu 610213, China

**Keywords:** pruning waste, *Cinnamomum camphora*, phytotoxicity, germination, nutrient absorption, root damage

## Abstract

Much previous research has indicated most composts of pruning waste are characterized by potential phytotoxicity, it is highly correlated with the chemical compounds of raw materials. *Cinnamomum camphora,* a common kind of pruning waste in Southeast Asia and East Asia, is characterized by intense bioactivities due to complex chemical components. This study investigated the potential phytotoxicity of *C. camphora* pruning waste in light of germination and higher plant growth. *C. camphora* extracted from leaves completely inhibited seed germination and still showed suppression of root elongation at an extremely low dosage. *C. camphora* extract also displayed significant inhibition of nutrient absorption in tomato seedlings, including moisture, available nutrients (N, P and K) and key microelements (Fe, Mn, Zn and S). The gene expression of aquaporins and transporters of nitrate and phosphate was significantly up-regulated in roots. This could be regarded as a positive response to *C. camphora* extract for enhancing nutrient absorption. Moreover, the severe damage to the plasma membrane in roots caused by *C. camphora* extract might seriously affect nutrient absorption. Camphor is the main component of the *C. camphora* extract that may induce the phytotoxicity of plasma membrane damage, resulting in the inhibition of nutrient absorption and low biomass accumulation. This study provided a new understanding of the ecotoxicological effects of *C. camphora* pruning waste, indicating that the harmless disposal of pruning waste requires much attention and exploration in the future.

## 1. Introduction

In the past decades, rapid urbanization has caused a remarkable increase in urban green space and the production of pruning waste, including grass clipping, tree prunings and leafy residues. Over 350 million tons of pruning waste is generated per year in China, according to the National Bureau of Statistics of China [1]. Composting is one of the most common approaches for pruning waste treatment, and the final compost can be used for land application [2]. However, the composting treatment of pruning waste has not made progress in the past decades. Much previous research has indicated most final composts of pruning waste are characterized by potential ecotoxicity on seed germination and plant growth [3,4]. The potential phytotoxicity showed the highest normalized impact potentials for all the scenarios and was unaffected by the different garden waste treatments [5]. These results suggested that pruning waste has been converted to composts with phytotoxicity present. Moreover, the phytotoxicity of pruning waste is highly correlated with the source and species of raw materials [4,6]. Therefore, it is necessary to evaluate the potential phytotoxicity of typical species for urban landscaping.

*Cinnamomum camphora* is one of the most important and widely used evergreen species for urban landscaping in Southeast and East Asia. *C. camphora,* as a border tree, accounts for more than 50% of decorative trees in many cities of southern China [7]. There is plenty of *C. camphora* waste leaves produced by pruning, which has been disposed of by traditional composting along with other pruning waste. However, many compounds from *C. camphora* leaves significantly exhibit antifungal and antibacterial activities [8,9,10]. Pinoresinol from *C. camphora* leaves inhibited the growth of *Escherichia coli*, *Pseudomonas aeruginosa*, *Staphylococcus aureus* and *Salmonella enterica* [11], decreasing the transmission risk of pathogenic bacteria. However, camphor and pinene of *C. camphora* extract exhibited antifungal activity against *Aspergillus niger* and *Bacillus*, which contribute to the degradation of organic matter during composting [12]. The antifungal activity of these compounds causes weak biological activities during composting, such that chemicals and derivates cannot be degraded to innoxious substances and be reserved in the final compost [6,13]. Moreover, the water extract of *C. camphora* leaves showed significant inhibition of microalgal growth by photosynthetic pigment degradation [13,14]. Therefore, it is necessary to evaluate the phytotoxicity of *C. camphora* pruning waste due to its potential treatments and land application. This study investigated the phytotoxicity of *C. camphora* water extract on seed germination and tomato seedling growth and analyzed the negative effect on root growth and nutrient absorption. This work would enhance the understanding of the potential ecotoxicity of *C. camphora* pruning waste during the treatment process.

## 2. Materials and Methods

### 2.1. Preparation of C. camphora Extract

Chemical components of *C. camphora* pruning waste are released into the water and migrate into the plant rhizosphere during land application. This study aimed to investigate the potential phytotoxicity of *C. camphora* water extract. *C. camphora* water extract was obtained according to the classic method with a ratio of 1:10 (*w*/*v*). This method has been applied to many national standards for evaluating the quality of plant media in China. *C. camphora* pruning waste was collected from the Chengdu Academy of Agriculture and Forestry Sciences in Sichuan province. Fresh leaves were dried at 60 °C and smashed with a pulverizer (<2 mm). The pulverized materials of 10 g were extracted with 100 mL of deionized water at 25 °C for 2 h [14]. The water extract solution was centrifuged at 5000 rpm for 10 min and sent through 0.22 μm membrane filters at a concentration of 100 mg/mL.

### 2.2. Seed Germination

The *C. camphora* extract was diluted by distilled water to concentrations of 5 mg/mL and 20 mg/mL. Separately, 5 mL of the diluted extract and deionized water (control) were added to 9 cm square dishes containing 20 seeds of Chinese cabbage, which were covered with one germinating and one filter paper. The seeds’ germination was performed at 25 °C for 72 h in darkness. After this period, the number of seeds germinated was counted, and the radical length was measured. The germination index (GI) was calculated by the following equation:

(1) $$\mathrm{GI}\;(\%)=\frac{\mathrm{seed}\;\mathrm{germonation}\;(\%)\;\times \mathrm{root}\;\mathrm{length}\;\mathrm{in}\;\mathrm{treatment}}{\mathrm{seed}\;\mathrm{germonation}\;(\%)\;\times \mathrm{root}\;\mathrm{length}\;\mathrm{in}\;\mathrm{control}}\times 100$$

To investigate the effects of the pH value, conductivity (EC) value and chemical compounds of *C. camphora* extract on seed germination, deionized water samples were prepared and characterized by the same pH and EC values of the *C. camphora* water extract (100 mg/mL). MgSO_4_·7H_2_O (2.5 g/L) and H_2_SO_4_ (1 mmol/L) were used to adjust the pH (5.5) and EC (0.8 mS/cm) of the deionized water, and the seed germination process was also performed.

### 2.3. Tomato Seedling Growth with C. camphora Extract

Tomato seedlings were cultivated in 50% strength of Hoagland’s solution for 12 days. Subsequently, seedlings with a similar size (fresh weight: 2 ± 0.2 g) were washed three times with deionized water and transferred into new Hoagland’s solution (50%), which contained 5 mg/mL and 20 mg/mL of *C. camphora* extract, respectively. Hoagland’s solution details are shown in Table S1. The plant biomass was collected at the end of 14 days of cultivation. The fresh weight of the entire plant, root and shoot was measured.

### 2.4. Moisture and Nutrients Absorption of Tomato Seedlings

Moisture uptake was measured by recording the volume of the solution at the start and end of experiments. Available nutrients (N, P and K) uptake was calculated according to the following format:

(2) $$nutrient\;uptake\;(mg)=(C_{1}\times V_{1})-(C_{2}\times V_{2})$$

*C*_1_, the concentration of elements in solution;
*V*_1_, the total volume of solution for 14 days of cultivation;
*C*_2_, the residual concentration of elements in residual solution;
*V*_2_, the residual volume of solution at the end of 14 days of cultivation;

Roots and leaves of tomato seedlings were dried and ground. Approximately 50 mg of ground samples were digestated by concentrated H_2_SO_4_ and H_2_O_2_. The digestate solutions were filtered using a 0.22 μm membrane, and the content of B, Ca, Mg and microelements were measured by inductively coupled plasma optical emission spectroscopy (ICP-OES) [15].

### 2.5. Root Plasma Membrane Integrity

Contaminant-exposed roots were washed three times with deionized water and incubated in 0.025% Evan’s blue solution (10 mL, pH 5.6) for 30 min to detect the plasma membrane integrity. The Evan’s blue-stained roots were washed with 0.01 mM PBS buffer solution three times and analyzed using a scanner (Perfection V39, EPSON, USA). The blue coloring indicated the degree of damage to the plasma membrane in the root.

### 2.6. Determination of Abscisic Acid (ABA) and Malonaldehyde (MDA)

Root tissue weighing 0.2 g was homogenized by 1.8 mL of PBS solution at 4 °C. The supernatant was obtained through centrifugation at 3000 rpm (20 min, 4 °C). The contents of ABA and MDA were measured using ABA ELISA and MDA ELISA kits following the manual.

### 2.7. Gene Expression

After exposure to the *C. camphora* extract for 14 days, the roots were washed three times with deionized water and frozen in liquid nitrogen. The roots were subsequently ground before RNA isolation and analysis for gene expression of phosphate transporters, nitrate transporters and aquaporins such as plasma membrane intrinsic protein (PIP), tonoplast intrinsic protein (TIP) and Nod26-like intrinsic protein (NIP). The extraction of total RNA from roots, evaluation of the quality and quantity of extracted RNA samples, reverse transcription of extracted RNA and the quantification of gene expression were performed. Specific primers are provided in Table S2.

### 2.8. Component Analysis of C. camphora Water Extract

The 100 mg/mL *C. camphora* extract was freeze-dried using a vacuum lyophilizer (SCIENTA-12ND, China). The freeze-dried powder was analyzed by pyrolysis gas chromatography-mass spectrum (PyGC-MS) according to the modified method [16,17]. The temperature of the pyrolysis increased from 30 °C to 350 °C at a ratio of 20 °C/min and was maintained for 10 s. The pyrolysis products were analyzed online and linked GC/MS (QP2010 Ultra) with a neutral phase. The GC was run with a 30 m × 0.25 mm × 0.25 μm HP-5MS capillary column. The carrier gas was helium, and the injection port temperature was 250 °C. The temperature of the column was programmed to increase from 50 °C to 180 °C at a rate of 20 °C/min, then increase to 250 °C at a rate of 10 °C/min and maintained for 15 min. The mass spectrometer ionization voltage was 70 eV, with a scan range of 30–660 m/z for all samples analyzed.

### 2.9. Statistical Analysis

Data were presented as mean ± standard deviation. All the treatments contained at least three replicates. Statistical analysis of one-way ANOVA was performed with Origin software.

## 3. Results and Discussion

### 3.1. Inhibition of C. camphora Extract on Seed Germination

**Figure 1:**
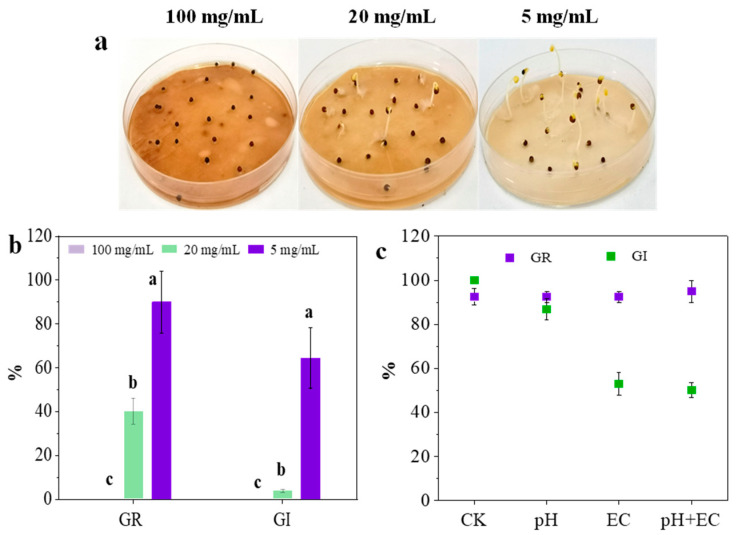
Seed germination of *C. camphora* extract (**a**,**b**) and deionized water samples (**c**). Deionized water samples are characterized by the same pH (5.5) and EC (0.8 mS/cm) value in 100 mg/mL of *C. camphora* extract. (The significant differences among different treatments are marked with different letters, *p* < 0.05).

The value of the germination index (GI) was considered a traditional standard to evaluate the phytotoxicity of raw materials and final compost during the treatment process of agricultural solid waste. This study also evaluated the phytotoxicity of *C. camphora* pruning waste according to the GI and GR values. It was obvious that *C. camphora* extract (100 mg/mL) showed complete inhibition with no seed germination (Figure 1a,b). Furthermore, the GR and GI values showed a significant increase with the reduction of the concentration of *C. camphora* extract. However, the GI value only increased to 64%, while the GR value was 90% when exposed to *C. camphora* extract of 5 mg/mL. The GI value was lower than the non-phytotoxic standard of 80%. This implied that a low dosage of *C. camphora* extract still showed a negative effect on root elongation. The complete inhibition of seed germination indicated that *C. camphora* pruning waste must be treated seriously. Moreover, the strong inhibition of root elongation caused by a low dosage of *C. camphora* extract indicated that the treatment of mixed pruning waste also concerns the content of *C. camphora* waste.

pH and EC have been considered the main factors causing the inhibition of seed germination and root elongation in many compost studies [18]. Little research focuses on the roles of chemical components from raw materials. In this study, to analyze the effect of pH, EC and chemical components on seed germination, deionized water samples characterized by the same values of pH (5.5) and EC (0.8 mS/cm) in a *C. camphora* extract (100 mg/mL) were prepared, and the GR values and GI values of these samples were measured. As shown in Figure 1c, the seed germination was not affected by pH and EC (GR > 90%). However, the GI value of deionized water with an equal EC was significantly lower than that of deionized water with an equal pH (*p* < 0.01). Compared with the results of three deionized water samples in seed germination, it was found that EC in *C. camphora* extract had a negative effect on root elongation rather than pH. In addition, the GI value of deionized water samples with the same condition of pH and EC was up to 50.16%. However, there was no GI value of *C. camphora* extract (100 mg/mL) due to the complete inhibition of seed germination (Figure 1a,b). This indicated that the chemical components of a *C. camphora* extract might be the crucial factor for inhibiting seed germination and root elongation. A previous study found that the germination index (GI) of green waste dominated by *C. camphora* significantly improved following torrefaction while decreasing the total alkaloid and flavonoid [1]. These results implied the phytotoxicity of chemical components from *C. camphora* pruning waste.

### 3.2. Effect of C. camphora Extract on Tomato Seedlings Growth

#### 3.2.1. Growth Inhibition

**Figure 2:**
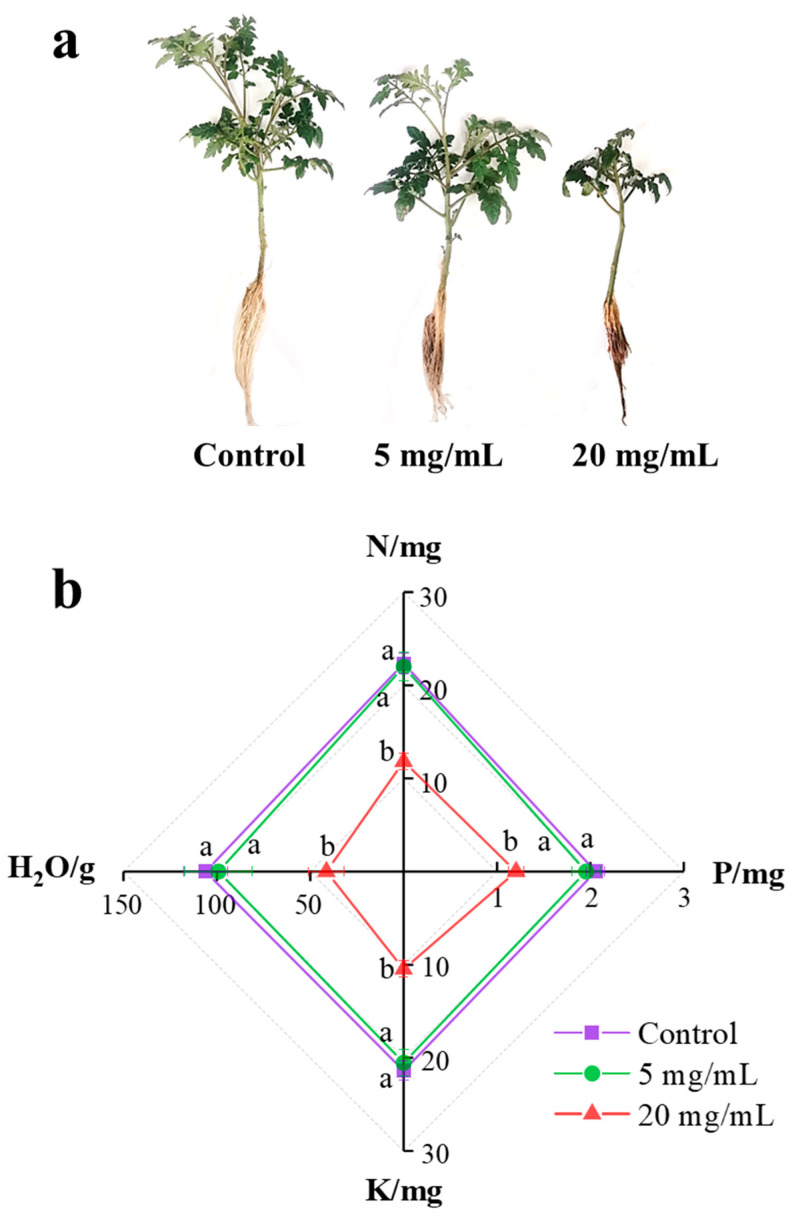
The growth (**a**) and available nutrient (**b**) (N, P and K) absorption of tomato seedlings after exposure to a *C. camphora* extract for 14 days. (The significant difference among treatments is marked with different letters, *p* < 0.05).

After a 14-day exposure to *C. camphora* extract, it was obvious that the growth of tomato seedlings was significantly inhibited. As shown in Figure 2a, the height and number of leaves of the plant obviously declined with the concentration of *C. camphora* extract increasing. After exposure to *C. camphora* extracts of 5 and 20 mg/mL, the fresh biomass of the plant reduced from 8.06 ± 0.37 g in the control to 6.45 ± 0.87 g and 4.52 ± 0.67 g (Table 1), respectively. Moreover, the elongation of the root was obviously inhibited by *C. camphora* extract according to the significant difference in root length (*p* < 0.05). The fresh biomass of the shoot, when treated with *C. camphora* extracts of 5 and 20 mg/mL reduced by 24.73 and 55.60%, respectively. These results indicated that *C. camphora* extract might inhibit the nutrient absorption in the root, resulting in less biomass than control.

The extract of fresh *C. camphora* leaves at 5 and 20 mg/mL killed 80% of *Microcystis aeruginosa* and *Chlamydomonas reinhardtii* cells after being treated for 48 h [14]. As the main component of *C. camphora* extract, camphor killed the whole cells of *C. reinhardtii* only at 2.4 mM after 48 h and of α-terpineol and linalool after only 24 h [15]. Higher plants generally show better tolerance to abiotic stress than lower plants like microalgae. However, a *C. camphora* extract of 5 mg/mL still showed significant inhibition of tomato growth. Overall, *C. camphora* extracts showed serious phytotoxicity to tomato seedling growth.

#### 3.2.2. Inhibition of Moisture and Nutrient Absorption

As shown in Figure 2b, *C. camphora* extracts produced a significantly negative effect on the absorption of moisture and available nutrients (N, P and K) at the concentration of 20 mg/mL (Figure 2b). Compared with control, the absorption of moisture, N, P and K was inhibited at ratios of 61.37, 47.11, 41.75 and 50.94%, respectively. As shown in Table 2, the absorption of Fe, Mn, Zn and S also showed significant suppression under exposure to a *C. camphora* extract (*p* < 0.05). As for Fe and Mn absorption, there was a more significant distinction in the root than leaves (*p* < 0.05). After a 14-day exposure to *C. camphora* extracts of 5 and 20 mg/mL, Fe content in the root decreased by 48.08 and 69.23%, respectively. Mn content in the root similarly decreased by 23.08 and 67.30%, respectively. However, the inhibition of Zn and S absorption was only found in the root, and their contents were reduced by 46.43 and 28.70%, respectively. To sum up, the inhibition of microelement absorption caused by *C. camphora* extract is as follows: Fe > Mn > Zn > S. Fe, Mn and Zn maintain the activities of various enzymes, which play roles in many physiological processes, including photosynthesis, aerobic respiration, nitrogen fixation, redox reaction and antioxidation [15,19]. Previous studies reported S greatly contributes to protein biosynthesis related to the detoxification pathway of heavy metals [20]. Therefore, decreases in the content of Fe, Mn, Zn and S may reflect the weak resistance of seedlings against *C. camphora* extracts. In addition, *C. camphora* extracts did not influence the absorption of Mg, Ca and B due to similar content of roots and leaves among the three groups.

#### 3.2.3. Gene Expression of Nutrients Transporters

**Figure 3:**
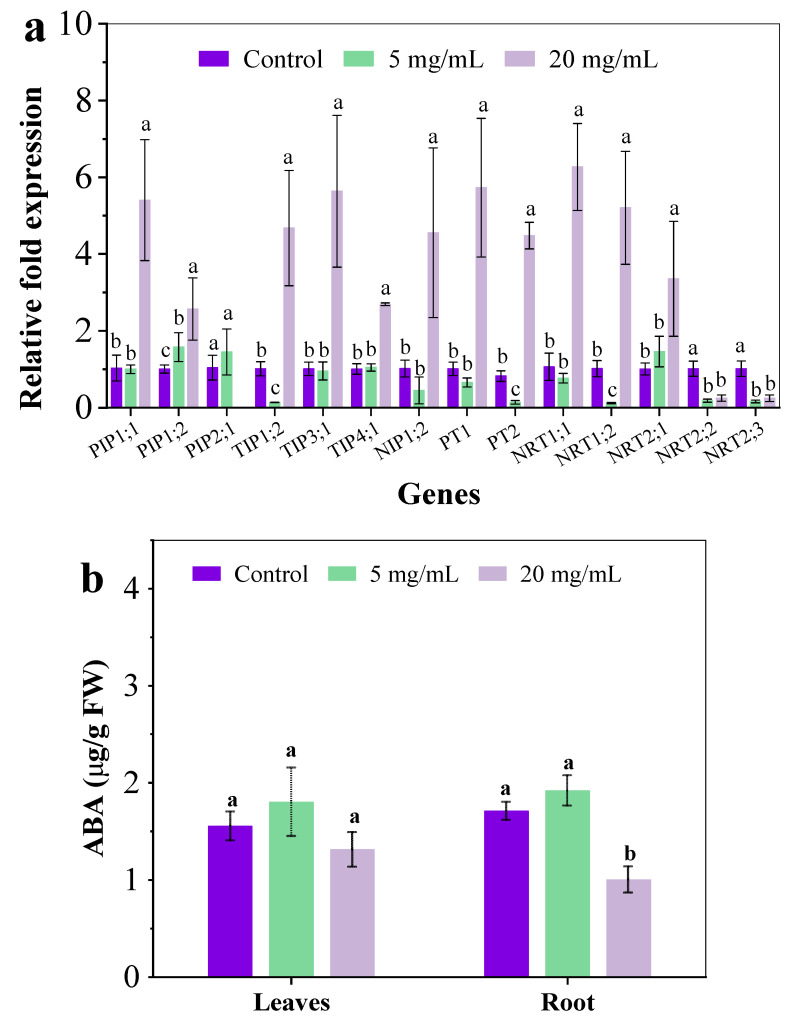
The relative gene expression in roots (**a**) and the ABA content (**b**) after exposure to *C. camphora* extract for 14 days. (The significant difference among different treatments is marked with different letters, *p* < 0.05.)

As shown in Figure 2b, *C. camphora* extracts significantly inhibited the absorption of moisture, N and P. Aquaporins are water channel proteins that facilitate and regulate the passive movement of water molecules down a water potential gradient. Many studies have indicated the important role of aquaporins in defense and resistance under abiotic stress [21]. The expression of genes associated with aquaporins, including plasma membrane protein (PIP), tonoplast intrinsic protein (TIP) and Nod26-like intrinsic protein (NIP) related genes, were analyzed. After exposure to *C. camphora* extracts for 14 days, the expression of five genes showed no obvious differences between the control and group treated with 5 mg/mL except PIP1;2 and TIP1;2. However, the expression of PIP1;1, PIP2;1, TIP1;2, TIP3;1, TIP4;1 and NIP1;2 was significantly increased by 4.25, 1.56, 3.61, 4.57, 1.67 and 3.48-fold when exposed to 20 mg/mL of *C. camphora* extract, respectively (Figure 3a). ABA is an important abiotic stress-related phytohormone that can down-regulate the level of aquaporin-related genes [22]. The ABA concentration in roots treated with 20 mg/mL *C. camphora* extract was significantly reduced by 41.18% as compared to the control (Figure 3b). Therefore, the up-regulated aquaporin-related genes in the root treated with 20 mg/mL *C. camphora* extract could be attributed to the decreased ABA content and the less root area. Aquaporins are known to be water channel proteins that exhibit numerous functional properties in plant growth and development, such as stress response, nutrient absorption and transportation (N, B, CO_2_) [23]. This indicated that the up-regulated expression of aquaporin genes in the root could be attributed to a positive response to resisting the abiotic stress from *C. camphora* extract. Furthermore, this study also found that the expression of genes associated with phosphate transporters and nitrate transporters was also up-regulated when exposed to 20 mg/mL *C. camphora* extracts (Figure 3a). The expression of PT1, PT2, NRT1;1. NRT1;2 and NRT2;1 was significantly increased by 4.67, 4.45, 4.89, 4.12 and 2.32-fold, respectively. However, the expression of NRT2;2 and NRT2;3 was decreased as compared with the control. This indicated that NRT1;1, NRT1;2 and NRT2;1 played an important role in resisting the inhibition of nitrogen absorption caused by *C. camphora* extract. Overall, the up-regulated gene expression associated with the absorption of moisture, N and P could be regarded as a positive response to resisting *C. camphora* extract by enhancing nutrient absorption.

### 3.3. Plasma Membrane Damage in Roots

**Figure 4:**
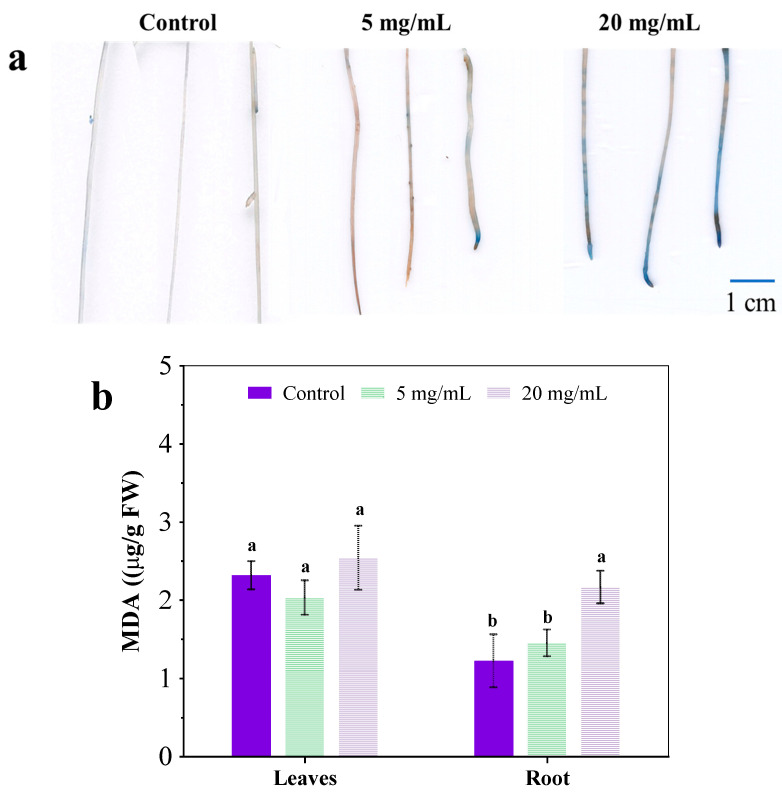
Root stained by Evan’s blue (**a**) and MDA content (**b**) after exposure to a *C. camphora* extract for 14 days. (The significant difference among different treatments is marked with different letters, *p* < 0.05.)

Many previous reports have indicated the plasma membrane damage in the root under abiotic stress, such as heavy metals alone or co-exposure with graphene oxide [24,25]. Graphene oxide-enhanced oxidative stress is caused by As and Ag in the root of a plant, resulting in the loss of cell integrity, the inhibition of nutrient absorption and the compromise of key detoxification pathways such as complexation with glutathione and efflux [15,26,27]. In this study, Evan’s blue staining was used to investigate the plasma membrane integrity in exposed roots and the extent of blue coloring directly correlated with membrane damage [27]. In Figure 4a, some slight blue color was evident in the roots of the control group and treatment group with 5 mg/mL extract, suggesting that plasma membranes in the root were largely intact. After exposure to 20 mg/mL extract, the blue coloring in the root was visibly darker than the other two, indicating that the addition of *C. camphora* extracts compromised the root’s plasma membrane. Moreover, *C. camphora* extract also resulted in severe lipid peroxidation in roots, as indicated by an increased malondialdehyde (MDA) content (Figure 4b). Severe damage to the root would affect the structure and function of the plasma membrane, resulting in negative effects on physiological processes such as ion homeostasis, osmotic pressure and nutrient assimilation [15,28]. Moreover, the expression of aquaporin, phosphate transporters and nitrate transporters were up-regulated while showing the significant inhibition of moisture, N and P absorption (Figure 2b and Figure 3a). This may be an essential mechanism for the structure and function of transporters and channels on the plasma membrane in the root that was destroyed by chemical components of *C. camphora* extract, resulting in poor nutrient absorption and low biomass accumulation.

### 3.4. Component Analysis of C. camphora Extract

*C. camphora* is a typical greening tree species, the unique smell of which is attributed to volatile components that show the inhibitory activities of bacteria, fungi and algae growth [9,12]. The component analysis of *C. camphora* extract was performed and compared with previous studies (Table 3). Currently, there are five different chemotypes observed worldwide for *C. camphora*: camphor, linalool, eucalyptol, nerolidol, and borneol [12,29]. As shown in Table 3, it was indicated that camphor was the main component of *C. camphora* water extract in this study. The species of *C. camphora* in China mainly includes the two chemotypes, camphor and linalool. Specifically, the camphor content of crude water extract was up to 66.10% in a previous study [30]. In this study, camphor was approximately up to 50%, and followed by ethylene-glycol (28.2%), quinic acid (3.93%), acetic acid (3.56%) and coumaran (1.27%).

There is much research proving the phototoxicity of *C. camphora* water extract. *C. camphora* extracts containing much camphor showed significant inhibition of seed germination and seedling growth of *Lactuca sativa* and *Lolium perenne* [12]. Aqueous extract from *Abies Alba*, also containing monoterpenes such as camphene, effectively inhibited tomato seed germination and radicle elongation [31]. Moreover, it was reported that *C. camphora* water extract caused significant inhibition of cell growth of *Microcystis aeruginosa* and *Chlamydomonas reinhardtii*, and the whole cells were killed by camphor at 2.4 mM after 48 h [13,15]. As the main chemical components of *C. camphora* water extract, linalool, eucalyptol and acetic acid-induced the programmed cell death of *C. reinhardtii*, specifically resulting in H_2_O_2_ production, photosynthesis decrease, caspase-like activities, nuclear variation and DNA degradation [32,33,34]. In this study, *C. camphora* water extract also displayed a severe inhibitory effect on seed germination and tomato seedling growth. Camphor may be the main component inducing root damage and inhibition of nutrient absorption.

This study aimed to investigate the potential phytotoxicity of *C. camphora* pruning waste due to its characteristics on bioactivities. Bioactivities of plant extract can be attributed to chemical components, most of which can be classified into allelochemicals [35]. Allelopathy is a phenomenon observed in many plants that involve the production and release of bioactive compounds into the environment [36]. Many previous studies have reported that allelochemicals show intense inhibition in seed germination, radicle elongation and growth of neighboring plants [37,38,39]. It is known that *C. camphora* maintains survival advantages in the area by inhibiting other species’ growth via allelopathy [40]. The results in this study also confirmed the intense phytotoxicity of *C. camphora* pruning waste by seed germination and cultivation of the higher plant. Hence, we must consider the ecotoxicological effect of *C. camphora* pruning waste on plant growth because of its large yield, treating approach and land application.

## 4. Conclusions

This study confirmed the serious ecotoxicity of water extract of *C. camphora* pruning waste on seed germination and higher plant growth. *C. camphora* extract showed great inhibition of seed germination and root elongation and suppressed the nutrient absorption of tomato seedlings, including moisture, available nutrients and key microelements. Severe damage to the plasma membrane in the root caused by *C. camphora* extract may be responsible for the inhibition of nutrient absorption. It indicated that the treatment of *C. camphora* pruning waste requires more attention and research for alternative approaches for harmless disposal.

## Figures and Tables

**Table 1:** The growth of tomato seedlings after exposure to a *C. camphora* extract for 14 days.

Treatments	Fresh Weight			Height/cm	Root Length/cm
	Plant/g	Root/g	Shoot/g		
control	8.06 ± 0.37 ^a^	2.47 ± 0.40 ^a^	5.54 ± 0.59 ^a^	18.67 ± 2.14 ^a^	12.83 ± 2.77 ^a^
5 mg/mL	6.45 ± 0.87 ^b^	2.29 ± 0.23 ^a^	4.17 ± 0.89 ^b^	16.43 ± 1.53 ^a^	8.85 ± 0.55 ^b^
20 mg/mL	4.52 ± 0.67 ^c^	2.06 ± 0.19 ^a^	2.46 ± 0.75 ^c^	12.5 ± 1.45 ^b^	6.54 ± 0.98 ^b^

The difference among treatments is marked with different letters (*p* < 0.05).

**Table 2:** The content of nutrient elements in the roots and leaves of tomato seedlings after exposure to *C. camphora* extract for 14 days.

**Leaves**	**Fe (mg/g DW)**	**Mn (mg/g DW)**	**B (mg/g DW)**	**S (mg/g DW)**	**Zn (μg/g DW)**	**Ca (mg/g DW)**	**Mg (mg/g DW)**
control	0.43 ± 0.024 ^a^	0.11 ± 0.013 ^a^	0.03 ± 0.004 ^a^	5.54 ± 0.81 ^b^	0.18 ± 0.054 ^b^	4.17 ± 0.63 ^a^	3.93 ± 0.49 ^a^
5 mg/mL	0.23 ± 0.015 ^b^	0.12 ± 0.010 ^a^	0.04 ± 0.004 ^a^	7.34 ± 0.80 ^a^	0.14 ± 0.034 ^b^	4.70 ± 0.62 ^a^	3.85 ± 0.98 ^a^
20 mg/mL	0.25 ± 0.019 ^b^	0.09 ± 0.017 ^b^	0.025 ± 0.004 ^a^	4.31 ± 0.18 ^b^	0.48 ± 0.059 ^a^	4.26 ± 0.97 ^a^	4.94 ± 0.49 ^b^
**Root**	**Fe (mg/g DW)**	**Mn (mg/g DW)**	**B (mg/g DW)**	**S (mg/g DW)**	**Zn (mg/g DW)**	**Ca (mg/g DW)**	**Mg (mg/g DW)**
control	0.52 ± 0.053 ^a^	0.52 ± 0.050 ^a^	4.63 ± 1.37 ^a^	7.63 ± 0.48 ^a^	0.14 ± 0.02 ^a^	6.43 ± 0.95 ^a^	1.76 ± 0.43 ^a^
5 mg/mL	0.27 ± 0.014 ^b^	0.40 ± 0.041 ^b^	3.99 ± 0.94 ^a^	7.43 ± 0.48 ^a^	0.11 ± 0.01 ^a^	6.99 ± 0.47 ^a^	1.72 ± 0.28 ^a^
20 mg/mL	0.16 ± 0.023 ^c^	0.17 ± 0.028 ^c^	4.52 ± 0.73 ^a^	5.44 ± 0.24 ^b^	0.075 ± 0.01 ^b^	6.72 ± 1.13 ^a^	2.08 ± 0.41 ^a^

The difference among treatments is marked with different letters (*p* < 0.05).

**Table 3:** The chemical components of *C. camphora* water extract.

Sample	Source	Solvent (Ratio: *m*/*v*)	Extraction	Component (Content, %)	References
Fresh leaves	Chengdu, China	Distilled water (1:10)	Shaked at 25 °C for 2 h	Camphor (50.19); Ethylene-glycol (28.20) Quinic acid (3.93); Acetic acid (3.56); Coumaran (1.27)	This study
Fresh leaves	Zhejiang, China	Distilled water (1:10)	Shaked at 25 °C for 48 h	Linalool (53.71); α-Terpienol (24.53); Camphor (8.45) Trans-linalool oxide (4.48); Eucalyptol (1.74)	[13]
Fresh leaves	Jiangxi, China	-	Microwave extraction at 580 W for 23 min	Camphor (66.10); Eucalyptol (15.45) β-Sabinene (3.94); β-Germacrene (2.67); α-Pinene (2.46)	[30]
		Distilled water (1:5)	Hydro distillation for 4 h	Camphor (53.68); Eucalyptol (17.55) α-Pinene (5.04); β-Sabinene (3.77); β-Germacrene (3.68)	[30]
Fallen leaves	Zhejiang, China	Distilled water (1:10)	Shaked at 25 °C for 48 h	Camphor (34.35); Linalool (26.31); α-Terpienol (5.24) 6-epi-Shyobunol (8.31); Hotrienol (5.11)	[14]
Fresh leaves	Hetauda, Nepal	Distilled water	Hydro distillation for 4 h	Camphor (36.5); Camphene (11.7) α-Pinene (7.7); β-Pinene (6.3); Sabinene (6.3)	[12]
Fresh leaves	Suzhou, China	-	Hydro distillation for 6 h	Camphor (40.54); Linalool (22.92) Cineole (11.26); p-Menth-1-en-8-ol (2.3) 3,7,11-Trimethyl-3-hydroxy-6,10-dodecadien-1-yl acetate (4.5)	[41]
Fresh leaves	Guangzhou, China	-	Hydro distillation for 2 h	Linalool (26.6); Eucalyptol (16.8); α-Terpienol (8.7) Isoborneol (8.1); β-Phellandrene (5.1)	[8]

## Data Availability

Not applicable.

## Supplementary Materials

The following supporting information can be downloaded at: https://www.mdpi.com/article/10.3390/ijerph191811617/s1. Table S1: The composition of 50% Hoagland’s solution for tomato seedling cultivation title. Table S2: Primers used in the investigation of gene expression.
