# Supplementary material for: Phytotoxicity of Chemical Compounds from Cinnamomum camphora Pruning Waste in Germination and Plant Cultivation
Source: Int J Environ Res Public Health. 2022 Sep 15;19(18):11617. doi: 10.3390/ijerph191811617 (PMC9517094; doi:10.3390/ijerph191811617)
Supplement: Supplementary file 1 [file ijerph-19-11617-s001.zip › ijerph-1877234-supplementary.pdf]

Phytotoxicity of Chemical Compounds from *Cinnamomum camphora* Pruning Waste in Germination and Plant Cultivation

Hong Wang [1,2,3], Wei Lin [1,3], Dongdong Zhang [1,3], Rui Yang [1,3], Wanlai Zhou [1,3] and Zhiyong Qi [1,3,*]

[1] Institute of Urban Agriculture, Chinese Academy of Agricultural Sciences, Chengdu 610213, China
[2] Biogas Institute of Ministry of Agriculture and Rural Affairs, Chengdu 610041, China
[3] Chengdu National Agricultural Science and Technology Center, Chengdu 610213, China
* Correspondence: qizhiyong@caas.cn

Table S1 The composition of 50% Hoagland's solution for tomato seedling cultivation

Component	mg/L		Component	g/L
$Ca(NO_3)_2 \cdot 4H_2O$	800	Fe-EDTA solution	$FeSO_4 \cdot 7H_2O$	5.6
KNO_3	300		Na-EDTA	7.5
$MgSO_4 \cdot 7\ H_2O$	220	Trace metal solution	H_3BO_3	2.8
KH_2PO_4	60		$MnCl_2 \cdot 4H_2O$	1.8
Fe-EDTA solution	1.25 mL		$CuSO_4 \cdot 5H_2O$	0.1
Trace metal solution	1 mL		$NaMoO_4 \cdot 2H_2O$	0.025
			$ZnSO_4 \cdot 5H_2O$	0.2

Table S2 Primers used in the investigation of gene expression

**Gene name**	**Primer sequence（5’ - 3’）**
α-tubulin-F	TGAACAACTCATAAGTGGCAAAG
α-tubulin-R	TCCAGCAGA AGTGACCCAAGAC
SlPIP1;1-F	GAAATCTTAGTGAGTGAGTGAG
SlPIP1;1-R	ATGATGATAGTTCACCAGG
SlPIP1;2-F	TCCTATTTTGGCACCTCTTCC
SlPIP1;2-R	ATCCCATGCCTCGTCTTTG
SlPIP2;1-F	GTGCTGCTGTTGTTTATGGACA
SlPIP2;1-R	CATCCAACACAACTCTAACAAC
SlTIP1;2-F	GTAGGAGGTCACATTACACT
SlTIP1;2-R	CGCACCACCAGCCAAGATAT
SlTIP3;1-F	TCACCACGAAAAGGGGCTAC
SlTIP3;1-R	CGAACTAAATACAATCACCATAGGC
SlTIP4;1-F	ATGGAGTTTTTGAGTCTTCTGC
SlTIP4;1-R	GCTGCGTTTCTGGCTTAGG
SlNIP1;2-F	GGTGATGCTAGAGACGGAGC
SlNIP1;2-R	TGACGGTAAGTCCCCAAACG
SlPT1-F	TTATACGCTGCTCAGCCCAC
SlPT1-R	TTCCCCTTCGTTTTCCCTCG
SlPT2-F	TGTGCCAGAGCCAAAAGGAA
SlPT2-R	ACCATTTTACAATAACACACTTGGC
NRT1;1-F	TTCAGCTCGGTTCTCGTGAC
NRT1;1-R	CAACAAGTACGGCCACAAGC
NRT1;2-F	TCCTTACCGATCTTCCCCGT
NRT1;2-R	CAAGCCGGAGTCTTTAGCCA
NRT2;1-F	GGGCTCACACAACTCCTCTT
NRT2;1-R	CACCCTCTGACTTGGCGTT
NRT2;2-F	TCAAGGGAACGGAAGAACATTATTA
NRT2;2-R	GCTCATTGAACTAAAGATTGACGAT
NRT2;3-F	CCTTCGGTGGTTACGCTTCT
NRT2;3-R	GGACCCGAAGTTTCCTCCTG
